# On the evolution of the serotonin transporter linked polymorphic region (5-HTTLPR) in primates

**DOI:** 10.3389/fnhum.2013.00588

**Published:** 2013-11-19

**Authors:** Seth D. Dobson, Lauren J. N. Brent

**Affiliations:** ^1^Department of Anthropology, Dartmouth CollegeHanover, NH, USA; ^2^Duke Institute for Brain Sciences and Center for Cognitive Neuroscience, Duke UniversityDurham, NC, USA

**Keywords:** serotonin transporter gene, group living, balancing selection, humans, macaques

## Abstract

Some allelic variants of the serotonin transporter linked polymorphic region (5-HTTLPR) result in lower levels of expression of the serotonin transporter gene (*SLC6A4*). These low-expressing (LE) alleles are associated with mental-health disorders in a minority of humans that carry them. Humans are not the only primates that exhibit this polymorphism; other species, including some monkeys, also have LE and high-expressing (HE) variants of 5-HTTLPR. We propose a behavioral genetic framework to explain the adaptive evolution of this polymorphism in primates, including humans. We hypothesize that both LE and HE alleles are maintained by balancing selection in species characterized by short-term fluctuations in social competition levels. More specifically, we propose that LE carriers benefit from their hypervigilant tendencies during periods of elevated competition, whereas HE homozygotes cope best when competition levels do not deviate from the norm. Thus, both alleles have long-term benefits when competition levels tend to vary substantially over relatively short timescales within a social group. We describe this hypothesis in detail and outline a series of predictions to test it. Some of these predictions are supported by findings in the current literature, while others remain areas of future research.

## Introduction

Understanding the neurobiological mechanisms that shape the production of behavior is a fundamental goal of neuroscience. Thanks to recent advances in genomics, it is now possible to investigate this question at the genetic level. A genetic variant that has received considerable attention in recent years is the serotonin transporter linked polymorphic region (5-HTTLPR), which is a promoter sequence that regulates the expression of the serotonin transporter gene (*SLC6A4*) (Canli and Lesch, 2007; Homberg and Lesch, 2011). Serotonin transporter (5-HTT) proteins mediate the reuptake of serotonin from the synaptic cleft, which serves to terminate neurotransmission and replenish serotonin stores in presynaptic terminals. *SLC6A4* expression is hypothesized to influence cortical development and consequently cognitive function, especially with regard to emotion regulation networks (Jedema et al., 2010).

Humans have two common versions of 5-HTTLPR, a “short” (S) allele, which consists of 14 tandem repeats, and a “long” (L) allele, which consists of 16 tandem repeats (Nakamura et al., 2000). There is geographic variation in the degree to which the L allele is more frequent than the S allele (Chiao and Blizinsky, 2010), and the latter is typically associated with lower quantities of 5-HTT resulting from reduced rates of *SLC6A4* transcription (Greenberg et al., 1999). However, some rare versions of the L allele, i.e., those characterized by additional single nucleotide mutations, also result in reduced amounts of 5-HTT (Hu et al., 2006). Given this complexity, we use the terms “low-expressing” and “high-expressing” to refer to functional variants of 5-HTTLPR.

The negative consequences of carrying low-expressing (LE) 5-HTTLPR alleles have been well documented (Caspi et al., 2010). For example, LE-allele carriers tend to score higher on personality tests that measure neuroticism, which is a risk factor for anxiety and depression (Lesch et al., 1996; Munafo et al., 2009). LE alleles do not necessarily result in mood disorders, however. Instead, environmental factors have been proposed to mediate the phenotypic effects of 5-HTTLPR throughout the lifespan (Homberg and van den Hove, 2012). Typically LE-allele carriers who experience stressful life events have a higher risk of depression than less-stressed LE carriers (Caspi et al., 2003).

Because most studies of 5-HTTLPR have tended to focus on mental-health disorders, relatively little attention has been paid to the potential benefits of LE alleles (Belsky et al., 2007; Homberg and Lesch, 2011). This is a glaring gap in our understanding of serotonin transporter polymorphisms for two main reasons. First, most people who carry LE alleles do not develop mental-health disorders, and in fact LE-allele carriers often respond more positively to environmental enrichment than high-expressing (HE) allele carriers (Belsky et al., 2009). Second, LE alleles are found in relatively high frequencies (>10%) in all human populations (Chiao and Blizinsky, 2010). These numbers are too high to be explained by mutation and gene flow alone, suggesting instead that this allele has been maintained by natural selection. Yet, it is highly unlikely for an allele with purely negative consequences to be selectively maintained (Belsky et al., 2009; Homberg and Lesch, 2011).

In recent years, various benefits of the LE variant of 5-HTTLPR have been proposed. For example, LE carriers exhibit increased activity of the amygdala in response to emotionally relevant stimuli (Hariri et al., 2002; Caspi et al., 2010), a greater response of the HPA-axis to aversive stimuli (Gotlib et al., 2008; Mueller et al., 2010; Way and Taylor, 2010), and increased immune response, blood pressure, and epinephrine during stressful tasks (Ohira et al., 2009; Fredericks et al., 2010). These findings may explain why LE carriers have difficultly disengaging from negative or threatening stimuli, and why they respond more strongly to both negative and positive environmental cues (Homberg and Lesch, 2011). LE-allele carriers are also better able to change their responses in line with shifts in reward context, and have been described as more cognitively flexible (Vallender et al., 2009; Jedema et al., 2010). Yet, despite this flexibility, LE carriers generally demonstrate an aversion to risks in financial (Crişan et al., 2009; Kuhnen and Chiao, 2009) and social contexts (Watson et al., 2009). Taken together, these finding have led to the suggestion that LE-allele carriers are overly sensitive to external stimuli (Homberg and Lesch, 2011). Such “hypervigilance” may be moderately harmful in the day-to-day, but highly beneficial under circumstances that have major impacts on fitness, such as when life-threatening situations arise (Homberg and Lesch, 2011).

It is important to note that several studies of 5-HTTLPR have failed to replicate previously documented phenotypic associations. This is due in part to the fact that novel significant results are more likely to be published than failed replication attempts (Duncan and Keller, 2011), and initial findings of significant associations appear to have overestimated the true effect sizes (Munafo et al., 2008). Moreover, genome-wide association studies (GWAS) of human disease and personality have only infrequently identified 5-HTTLPR, or indeed other common genetic variants, as important loci (Flint and Munafo, 2013). While meta-analyses of published findings have found statistically significant associations between 5-HTTLPR and some phenotypes (Schinka et al., 2004; Sen et al., 2004; Munafo et al., 2008, 2009; Murphy et al., 2013), the amount of phenotypic variation attributed to the polymorphism is often less than 5%, which some authors have suggested is too small to be indicative of a causal factor in disease (Flint and Munafo, 2013).

A broader comparative perspective on 5-HTTLPR might help to mitigate some of these complexities. Humans are not the only primates to exhibit natural variation at this locus, nor are we the only primates for which the serotonergic system is important. Several species of monkey and all extant species of ape are polymorphic for 5-HTTLPR (Table 1). The taxonomic breadth of this polymorphism provides an excellent opportunity to generate and test hypotheses concerning the evolutionary pressures acting on this system, and to do so independently of the complexities of human neuropsychopathology.

**Table 1:**
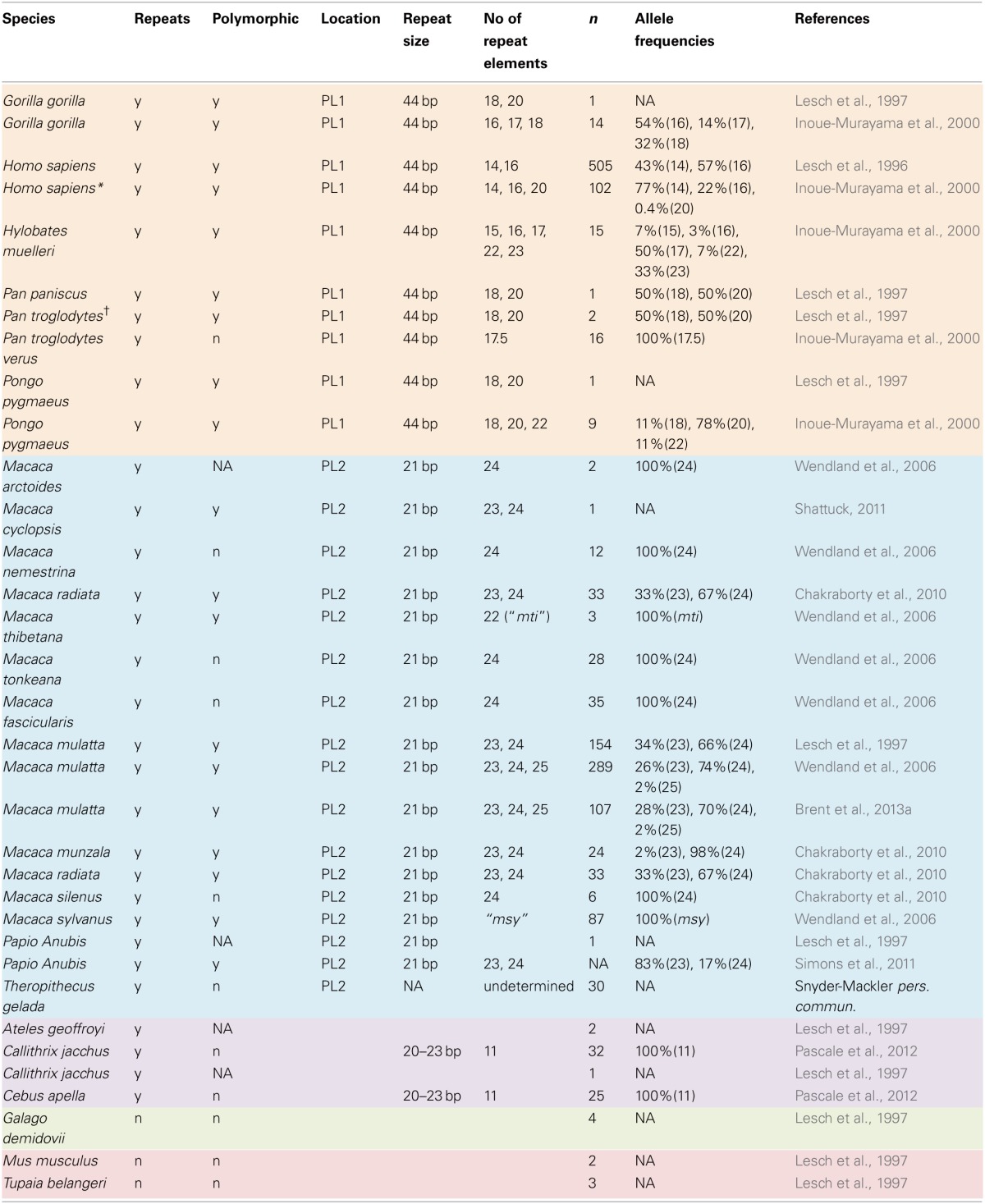
**Summary of 5-HTTLPR polymorphisms in primates and other species**.

*(orange, apes; blue, Old World monkeys; purple, New World monkeys; green, prosimians; red, non-primate mammals)*.

^*^Homo sapiens from Japan. ^†^Subspecies unknown. Location = polymorphic location.

The aims of this review are two-fold. First, we put forward an argument in favor of adopting an evolutionary perspective when studying 5-HTTLPR polymorphisms. Our intention is not to deny the important criticisms that have been raised regarding candidate genes studies. These are valid and should be taken into account when possible. However, we also believe that a broader, evolutionary perspective offers a valuable contribution to our understanding of this, and perhaps other common genetic variants. Second, we use this perspective to put forward a hypothesis for the evolution of 5-HTTLPR polymorphisms in primates. Our framework builds on previous (not entirely dissimilar) hypotheses and incorporates the most up-to-date findings regarding the primate serotonergic system. We make explicit links to primate social systems, and present an ecologically informed model that is generally applicable across the primate order. We conclude with a series of explicit predictions, some of which have already been supported by findings in the literature, while others remain areas for future research.

## An evolutionary perspective

### Mechanisms of polymorphism

A genetic polymorphism is defined as the presence of two or more alleles in a population at frequencies that are greater than expected by mutation and gene flow alone (Hedrick, 2009). Polymorphisms are actively maintained by balancing selection. This involves selection acting either through heterozygote advantage, frequency-dependence, niche divergence, or by the existence of two or more evolutionary stable strategies of roughly equal benefit. These mechanisms make very different predictions about the relationship between phenotypes and fitness. For example, under heterozygote advantage, carriers of one copy of the LE allele are predicted to do better than homozygotes for either the LE or HE alleles. This has important consequences for studies that lump LE homozygotes together with heterozygotes for the purpose of statistical analysis. Thus, any hypothesis that purports to explain the evolution of 5-HTTLPR polymorphisms must be explicit about the type of balancing selection that is implied.

### Small effect sizes

Natural selection acts on phenotypes not genotypes. This is because the relative fitness of a particular genotype depends on the benefits of the associated phenotype in a particular environment. If a beneficial phenotype is heritable at the population level, then selection will act to change allele frequencies over time. Phenotypic variation in a population does not have to be entirely, or even mostly, explained by genetic differences in order for natural selection to work. As long as there is a genetic association, even if it is relatively weak, selection acting on the phenotype will result in changes in allele frequencies.

This is an important point in light of the small effect sizes typically observed in genetic association studies of 5-HTTLPR. For example, in an early study of the relationship between 5-HTTLPR and anxiety-related traits, Lesch et al. (1996) observed that genotype explained only 3–4% of the total phenotypic variation in a large sample of 505 individuals. Subsequent meta-analyses have confirmed that the effects of LE alleles on individual differences in personality traits are relatively small (Schinka et al., 2004; Sen et al., 2004; Munafo et al., 2009). Similarly, early fMRI studies found that people with LE alleles tended to exhibit greater activation of the amygdala than HE-allele carriers (Hariri et al., 2002, 2005). But a recent meta-analysis of 31 imaging studies found that only 1% of the variance in amygdala activation was explained by genotype (Murphy et al., 2013). Thus, the “endophenotype” approach promoted by researchers in imaging genetics (Hariri et al., 2006) might not be the solution to the problem of small effect sizes in genetic association studies (Flint and Munafo, 2007).

Given the polygenic nature of complex traits, it is not surprising to observe small effects in association studies of isolated candidate genes. This is because multiple genes interact with each other, and the environment, to produce complex phenotypes. While the phenotypic effect of any given candidate gene may be relatively small, in reality the influence of genetics on complex behavioral phenotypes may be much larger. This is because many other genes that might be involved are usually not examined directly in genetic association studies that typically focus on one or two isolated candidate genes. Similarly, just as single candidate genes acting in isolation do not produce complex behavioral phenotypes, single endophenotypes do not produce complex behaviors either. Complex behavioral traits arise from complex neural networks, and each area of the brain involved may influence the phenotype in a small but essential way. Thus, small statistical effect sizes can belie the biological importance of a candidate gene or endophenotype because of the complexity of the system.

Lastly, the use of narrow means to quantify complex phenotypes might result in small effect sizes. Many behavioral traits are continua, with pathology residing at the extreme ends of trait distribution. However, for most traits non-pathological continuous variation constitutes the majority of observed variance. By exploring only disease outcomes, or by quantifying complex traits using data from a small number of experimental tasks, researchers run the risk of capturing only a small portion of the phenotypic variance, thereby reducing their ability to uncover meaningful genetic associations. Broader and more exhaustive characterizations of behavioral phenotypes, including those that aim to capture normal variation not just pathology, might help to solve this problem.

### Parsimony and the comparative method

The comparative method is a powerful tool for testing hypotheses about evolutionary convergence (Nunn and Barton, 2001). This approach models interspecific diversity as a series of natural experiments in the relationship between phenotypes and environments. When two or more species exhibit a similar phenotype, the comparative approach seeks to find a single adaptive explanation for every instance of convergence, rather than multiple species-specific explanations. This convention is an application of the principle of parsimony, which is the best place to start when formulating hypotheses about phenotypic similarities between species.

Tandem repeats in the 5-HTT promoter exist in all primates studied to date (Table 1), but not in species considered to be living analogues to the ancestor of primates, such as the tree shrew (Lesch et al., 1997). This suggests that repeats at this locus arose following the divergence of the primates from their common ancestor with other mammals. While a repeated element is found in the 5-HTT promoter of all primates, only some species express a variable number of repeats. For example, all tufted capuchin (*Cebus apella*) individuals have 11 repeats (Pascale et al., 2012). Variable numbers of repeats within the 5-HTT promoter occur at one of two known locations: polymorphic location number one (PL1), which is found in apes, and polymorphic location number two (PL2), which is found in Old World monkeys (Lesch et al., 1997). All species of ape genotyped to date (*n* = 5) are polymorphic at the promoter site, with repeat lengths ranging from 14 in humans, to 23 in gray gibbons (*Hylobates muelleri*) (Table 1). Most apes (hominoids) possess the 16-repeat HE allele (the “long” allele) along with a high prevalence of longer repeat lengths (18–20), whose impact on levels of 5-HTT expression are unknown (Lesch et al., 1997; Inoue-Murayama et al., 2000, 2008). Notably, humans are the only hominoid in which the LE 14-repeat allele has been found. In monkeys, 5-HTTLPR polymorphisms have been best characterized in the genus *Macaca*, the extant members of which are distributed mainly throughout Asia. The most common repeat lengths found in macaques are the shorter 23-length repeat, which is functionally analogous to the human LE allele, and the longer 24-length repeat, which is analogous to the human HE allele (Lesch et al., 1997). Of the 12 macaque species genotyped to date, five are polymorphic for the LE and HE alleles, while the rest are monomorphic for either the HE allele, or for a rare repeat of different length (e.g., the *msy* repeat found in *M. sylvanus*) (Table 1).

The presence of LE and HE 5-HTTLPR alleles throughout the primate order suggests that independent evolution has occurred multiple times at this locus. Thus, a strong argument can be made in favor of examining this genetic variant using a broad comparative approach. With this in mind, we have developed a behavioral genetic framework that attempts to explain the evolution of 5-HTTLPR polymorphisms in primates as a function of divergent strategies for coping with fluctuating levels of competition within groups.

## Behavioral genetic framework

### Social competition and 5-HTTLPR

Group living is beneficial for animals mainly because it reduces the risks of predation (Van Schaik, 1983). Yet, along with such benefits come certain costs, including competition between group members for access to mates and resources (Sterck et al., 1997). Many primates rely on social strategies to mitigate these costs (Kudo and Dunbar, 2001), and variation in sociality is associated with differential survival and reproductive success (Silk et al., 2003, 2010; Majolo et al., 2012; Brent et al., 2013a).

Some researchers have suggested that carriers of the LE allele are better able to mitigate the costs of within-group competition because they are more sensitive to social stimuli (Jansen et al., 2010; Heiming et al., 2011; Homberg and van den Hove, 2012). However, this statement implies that LE allele carriers are more successful than HE homozygotes in competitive societies. If this were true, then we would expect highly competitive species like rhesus macaques (*M. mulatta*) to be monomorphic for the LE allele, which is not the case. Therefore, we suggest that a new hypothesis is required that posits either a heterozygote advantage in competitive contexts, or that attempts to explain why both the LE and HE alleles might be similarly beneficial in the face of competition.

We propose the following framework; as with previous authors, we suggest that 5-HTTLPR is associated with an individual's ability to cope with intra-group competition. However, unlike previous authors that have focused on average differences in competition levels between species, or between groups of the same species (Wendland et al., 2006; Chakraborty et al., 2010), we suggest that the driving force underlying the evolution of this system is *variance in competition levels within a group over time*.

### Fluctuating competition levels over short periods of time

Physical and social environments are dynamic. Erratic changes can occur in the physical environment in the form of stochastic fluctuations in climate, predation pressure, and food availability. Similarly, variations in the social environment can result from demographic changes, breeding seasonality, and the varying demands of parental care (e.g., lactation and perceived infanticide risk). Any of these environmental changes can result in changes in the level of competition between members of a social group. These changes can occur over extended time periods (e.g., decades), but can also represent shorter periods, such as months or even days.

**Figure 1:**
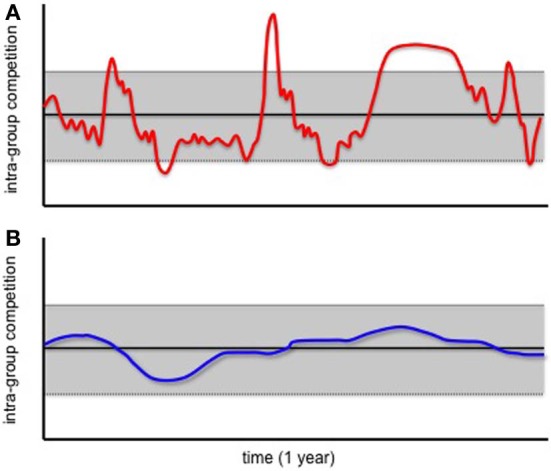
**Levels of intra-group competiton within hypothetical primate groups.** One group has highly variable levels of intra-group competetion over the period of 1 year **(A)**, the other has relatively invariable levels **(B)**. Black lines indicate mean intra- group competition levels, while gray bars indicate one standard deviation above and below the mean.

Figure 1 depicts how competition levels can fluctuate over a period of one year within a hypothetical primate group. In this example, competition levels do not deviate substantially from the mean most of the time. However, dramatic environmental changes occasionally result in competition levels that are substantially elevated (or substantially reduced) (Figure 1A). Given information on the extent to which competition levels vary over time, species can be classified into one of two groups: those for which competition levels are highly variable over short periods of time (Figure 1A), and those for which they are relatively stable (Figure 1B).

We propose that 5-HTTLPR polymorphisms evolve in primate species with levels of intra-group competition that are highly variable over short timeframes. As outlined in greater detail below, we hypothesize that LE-allele carriers cope best when intra-group competition levels are substantially elevated above average, whereas group members that are homozygous for the HE allele cope best when competition levels do not differ substantially from the norm.

### Differential sensitivity to competition

Rates of aggression are highest during periods of substantially elevated levels of intra-group competition (Brent et al., 2013b), making potentially fatal injuries more likely. Hypervigilance to social threats is likely to be beneficial in the face of elevated competition. Individuals can mitigate the risks of social aggression either by withdrawing from social interactions in general, or by continuing to engage socially while employing strategies to avoid conflicts. Commonly used conflict avoidance strategies include ritualized submissive gestures and low-cost signals of benign intent (Silk et al., 2000; Flack and de Waal, 2007). Risk avoidance is likely to be most adaptive during periods of elevated competition, when the potential benefits of risky behaviors are reduced relative to the costs of taking those risks.

LE-allele carriers tend to be risk averse and hypersensitive to both environmental stimuli and changes in reward context (Vallender et al., 2009; Jedema et al., 2010). As such, we propose that these individuals excel at attending to substantial fluctuations in competition levels and at adjusting their social strategies in response to those changes. Adjustments to social strategies that are likely to be beneficial during periods of elevated competition include heightened social vigilance and active avoidance of potentially hazardous social conflicts.

However, there are potential downsides to monitoring changes in the environment too closely. If small perturbations in the local environment do not reflect substantial changes in competition levels within the group as a whole, then it can be costly to monitor and respond to this type of random “noise.” Moreover, vigilance takes both time and energy, and interrupts other important behaviors, such as feeding (Chang et al., 2013). This in turn can reduce feeding efficiency and result in a reduction in total food intake. During periods in which competition levels do not deviate substantially from the mean, we propose that LE-allele carriers tend to waste time and energy monitoring and responding to relatively unimportant changes in their local environments. In contrast, because HE homozygotes are less responsive to fluctuations in competition levels in general, they can conserve time and energy when competition levels are not substantially elevated. This would give HE homozygotes an advantage over LE-allele carriers when environmental conditions are typical.

In Table 2, we summarize the behavioral “best practices” to cope with highly variable levels of intra-group competition. We predict that, due to their risk aversive tendencies and biased attention to social threats, LE-allele carriers will be best suited to situations in which competition levels are substantially elevated above average. In contrast, we predict that, due to their greater willingness to take (sometimes beneficial) risks, and their tendencies to conserve time and energy by not being overly vigilant or attending to minor changes in competition levels, HE homozygotes will be best suited to situations when competition levels are not substantially different from mean levels (Table 2).

**Table 2:** **Behavioral “best practices” in primates with fluctuating levels of within-group competition over time**.

	**Average competition levels**	**Elevated competition levels**
Social tendencies	Normal amounts of vigilance, occasionally engage in risky social interactions	Hypervigilance, strictly avoid risky social interactions
Sensitivity to changes in competition level	Ignore small fluctuations	Respond quickly to large fluctuations
Which genotype is better?	HE homozygotes	LE-allele carriers

Crucially, our hypothesis assumes that levels of intra-group competition are balanced over the lifetime of group members such that LE allele carriers and HE homozygotes have similar levels of long-term survival and reproductive success. It is for this reason that we have focused mainly on fluctuations in competition levels that occur over short timescales. Otherwise, balancing selection would not occur. It should also be noted that during periods of substantially reduced competition we expect selection pressures to be relaxed. In other words, all individuals cope well with periods of relative peacefulness, regardless of their behavioral tendencies. Finally, we would like to emphasize that highly variable levels of intra-group competition can occur in groups with both high and low baseline competition levels. For example, we have no reason to believe that substantial changes away from low levels of competition are less meaningful to group members than substantial changes in groups with relatively high baseline competition levels.

## Testing the predictions

### At the species level

To date, the only primates for which there is evidence of a functional 5-HTTLPR polymorphism are humans and five species of macaque (Table 1). It is not known whether the other species with this polymorphism exhibit differences in serotonergic functioning. Interestingly, humans and rhesus macaques (*M. mulatta*) are the two most widely distributed species of primate in the world, with humans occupying all continents, and rhesus macaques ranging from the Indian sub-continent, through the Himalayas to South-East Asian and China. The wide geographic range and behavioral flexibility of these two species have previously been linked to the presence of the LE allele (Suomi, 2006). However, the distributions of the other macaque species with the LE allele are relatively limited. Lion-tailed macaques (*M. silenus*), for example, are found only in a tiny section of Southern India (Molur et al., 2003). This suggests that geographic range size is not a good predictor of the presence/absence of the LE allele.

Alternatively, some researchers have argued that macaque species with less-tolerant social styles are more likely to have both LE and HE versions of 5-HTTLPR (Wendland et al., 2006; Canli and Lesch, 2007). However, this correlation has been rejected by more recent evidence of polymorphism among socially tolerant macaques (Chakraborty et al., 2010). One problem with the social style concept (Thierry, 2007) as applied to the question of 5-HTTLPR evolution is that it does not take into account variability in competition levels within social groups. Until classification schemes with explicit consideration of within-group variability are created, it will remain unclear whether intra-group competition levels are more variable in polymorphic compared to monomorphic species of macaque, or indeed other primates. The potential role of phylogenetic inertia (Blomberg and Garland, 2002) should also be considered in any interspecific analysis, as the genotypes of closely related species may be determined by their common ancestries more than their current socio-ecological conditions (Di Fiore and Rendall, 1994; Thierry et al., 2000).

Clearly there is also a general need for a greater understanding of 5-HTTLPR allele distribution and function across the primate order. Thus far, we have very little information about this promoter region in haplorhine primates outside of macaques, and we know almost nothing about this locus in strepsirhines. For many species that have been genotyped, sample sizes are often too small (e.g., *n* = 1) to definitively conclude whether the promoter is polymorphic or not (Table 1). Targeted genotyping of additional animals in a broader range of species will improve our understanding of this locus and its role in the evolution of primate behavior.

### At the individual level within species

Perhaps a more promising approach to testing our hypothesis is to examine the reproductive success of each genotype within species. However, fitness is challenging to measure in the best of circumstances, and this is especially true of long-lived animals that are slow to reproduce like primates. Nevertheless there are some tractable proxies, including number of offspring sired and subject morbidity. One study of free-ranging male rhesus macaques living on the island of Cayo Santiago, Puerto Rico, found that individuals with different 5-HTTLPR genotypes did not differ in the total number of offspring sired (Krawczak et al., 2005). That is, carriers of the LE allele had as much reproductive success as HE homozygotes. These findings suggest that balancing, rather than directional, selection is underway in the Cayo Santiago macaques, which supports our hypothesis. We can test this hypothesis further by examining differences in morbidity between individuals with different 5-HTTLPR genotypes. That is, we expect LE-allele carriers to receive fewer injuries during periods of elevated competition levels compared to HE homozygotes. This is because hypervigilance and high emotional reactivity in LE carriers should enable them to avoid aggressive encounters more effectively. In other words, the increase in morbidity associated with elevated competition levels should be greater in HE homozygotes than in LE-allele carriers.

Another approach would be to examine the response of the HPA-axis. Hormones, such as cortisol, are released in response to disruptions of homeostasis. This system triggers behavioral and physiological processes that help individuals to cope with stressors, and restore homeostasis (McEwen, 1998; McEwen and Seeman, 1999; Sapolsky, 2000; McEwen and Wingfield, 2003). Cortisol levels are therefore a good physiological indicator of how well individuals are coping with their current environments, with elevated baseline levels being indicative of frequent homeostatic disruptions. Due to their greater sensitivity to external stimuli, we predict that LE-allele carriers will have higher baseline cortisol levels compared to HE homozygotes, regardless of the competitive context. We also predict that LE-allele carriers will experience a more rapid increase in cortisol levels than HE homozygotes in response to increasing competition levels. In males, we may also expect a similar pattern for testosterone, with LE-allele carriers exhibiting a more rapid increase in testosterone levels compared to HE homozygotes in preparation for increased levels of competition. Data are currently being collected on Cayo Santiago to test these predictions in free-ranging rhesus macaques.

## Conclusions

Most research on 5-HTTLPR has emphasized the negative consequences of LE alleles under adverse environmental conditions. But the sheer prevalence of these so-called “risk alleles” within human populations, and among some non-human primates, suggests that LE-allele carriers enjoy a substantial amount of reproductive success. In this paper, we have outlined a detailed hypothesis for how 5-HTTLPR polymorphisms evolved in relation to ecologically relevant selective pressures in primates. It is clear that much more work needs to be done to test the predictions of our hypothesis. But we argue that the time has come for the fields of psychiatry and imaging genetics to take evolution more seriously.

### Conflict of interest statement

The authors declare that the research was conducted in the absence of any commercial or financial relationships that could be construed as a potential conflict of interest.
